# Some Popular Errors in the Treatment of Nervous Prostration1Read at the thirtieth annual meeting of the Medical Association of Central New York, held at Buffalo, October 19, 1897.

**Published:** 1898-05

**Authors:** B. C. Loveland

**Affiliations:** Clifton Springs


### SOME POPULAR ERRORS IN THE TREATMENT OF NERVOUS PROSTRATION.[1]

By B. C. LOVELAND, M. D., Clifton Springs.

FIRST, in reference to diagnosis. Apparently nearly all the functional neuroses characterised by deviations from the normal sensations and an inability, either real or imaginary, to do the usual quota of physical or mental work, are called neurasthenia or nervous prostration. I have seen cases called neurasthenia by prominent physicians when the physical condition of the patient was much more robust than that of the physician who made the diagnosis and the patient, too, was expending as much nervous energy, though foolishly, as the physician.

If neurasthenia is anything, it is a condition of weak nerves and weakness in anything implies an inability to perform its function. One so-called neurasthenic patient who complained sorely of inability to concentrate his mind and do his usual daily work wrote me twenty pages of well-written and consecutive description of his condition, as

1. Read at the thirtieth annual meeting of the Medical Association of Central New Yoık, held at Buffalo, October 19, 1897.

he termed it, telling all about his feelings to the very minutest detail and never once repeated himself. Now, such a patient has manifestly power for mental work and his trouble is not a lack of nerve power, neurasthenia, but a perversion of nerve power, hysteria.

An engine with all its parts in order and one hundred pounds of steam on has power and if the engineer turns the steam into the proper channel useful force will be exerted, *i. e.*, work will be done; if, however, he opens the escape valve he may allow all the steam to escape and he will have no power for work until a new head of steam has accumulated. A similar state of affairs was taking place with the so-called neurasthenic referred to. Nature is at work constantly to give him a reserve power, as one might say, and he is just as constantly wasting the energy given in self-study, worry and the like, thus throwing it away when it might exactly as well be put to some useful purpose, and that failure to properly direct his energy, not a lack of energy, constitutes his disease, if disease it may be called.

Another condition often confounded with neurasthenia is simple nervous exhaustion. Muscular exhaustion may be caused by muscular overstrain and in the same manner nervous exhaustion may be produced, but the amount of exercise, either muscular or nervous, required to produce exhaustion in either of the tissues respectively, cannot be determined in the abstract, as it varies with the physiological capital of the individual. Neurasthenia may be natural, *i. e.*, hereditary, or congenital, or it may be acquired. The natural neurasthenic is much more liable to nervous exhaustion than the person endowed with the ordinary amount of nervous capital, though nervous exhaustion may occur in the neurasthenic of either the natural or acquired variety, or in the person of normal endowment. This exhaustion, mistaken for neurasthenia, may bring great disaster to the patient.

The errors in treatment to which I would call attention are mainly due to the errors in diagnosis just mentioned, and consist principally in either filling the patient with tonics or stimulants and pushing him on to work, or allowing him to work on this false basis when he should have rest and nourishment; or putting him on a rest cure when he should have his brain cleared of some erroneous ideas and be kept at work.

The first class of cases referred to in this paper need to have their nerve force, which is being wasted, turned back into its proper channel. To that end they need some thoroughly competent physician to show them their physiological capital, prove to them that they have power and that their trouble consists not in a lack of power, as they suppose, but in a perversion of power. This task accomplished, they should be set at work at something, using work as their greatest safeguard, while the effort is still continued to efface from their minds the idea that they are neurasthenics. A man may be weaker than he thinks he is, but for practical purposes he is never stronger than he thinks he is. It is a frequent thing for me to see such patients who have been told that they had neurasthenia and must take a year off from business and perhaps take a trip to Europe and not even read or write letters. They usually accept the decision as coming from authority and become resigned to it as they would to typhoid fever, but when the year is past and they find that they are less able than at its beginning to do the ordinary mental work, whoever then has the task of setting them right has also the habit of thought and feeling of the whole year to overcome, for the self-study thus induced has become a great fascination. Numerous instances of various methods of handling this condition could be quoted, but that is not the object of this paper.

The same mistake is often made in cases of simple nervous exhaustion and the patient is either sent away for a hard trip from which he returns more exhausted or put to bed for from three to six weeks. In either case, if he has any tendency to hysteria or hypochondriasis, he has ample time to become a confirmed invalid by being deprived of his usual occupation, thus giving him all his time to devote to studying over his ailments. I do not mean to assail the "rest cure" so-called, or any other useful treatment, but to show how from a faulty application of the cure or method in properly selected cases, or its application to cases unsuited for it, it may be the means of developing extreme hysteria or at times melancholy. The convalescent or getting-up stage of the rest cure treatment deserves especial care and often more attention from the physician than any other time in the progress of the case.

The writer has had the difficult task, some score or more of times, of endeavoring to get a patient on his feet again after from one to four years or more of almost helpless invalidism, who owed a large share of his trouble to the popular treatment just mentioned, but which was not suited to the individual. The patient had nervous exhaustion, was told that he must lay off every thought of work and go to bed for a few weeks. He went to bed, hopeful that when the weeks were past and the disease had run its course he would get up well. He perhaps objected and was told that, although he did not now see how he could go to bed, in a week or two he would not hardly know how to get up, when once he had become relaxed. He found it even so. When the time had passed he found the getting up painful, became anxious and instead of setting his mind at rest by thinking that the pain was only caused by resuming activity in unused muscles, which was the truth, he thought, "Well! I must have been much worse than I thought, or even the doctor told me. Perhaps he was afraid to tell me for fear of discouraging me." The idea was not eradicated from his mind, he became hypochondriacal and thought if he was still so weak and sore on exertion he must be in need of more rest. He went to bed again for another rest, or, what was not much better, dragged about a miserable existence, walking as though he were treading on eggs and did not wish to break them, and fearing to write or speak above a whisper or low undertone, lest he should overtax his nerves. This state of affairs may last indefinitely and is at best very hard to break up and bring the patient back to anything like a normal individual. This is no ideal representation, but true to life, as it has come under my observation. Such a case was manifestly not a suitable one for rest cure,—or else the physician failed at the particular point alluded to as of special importance, the getting-up stage,—and was either hysteria to begin with, or nervous exhaustion which was turned into hysteria by being allowed to follow his own mental drift, and having nothing else to occupy his attention and thought than his own sensations.

In contradistinction to this, another case may show how a person with similar tendencies, if taken in time, may be soon restored to usefulness: A clergyman, 36 years old, of large physique and healthy appearance, consulted me March, 1897. He complained of insomnia, distressing pressure in his head, pain in the back of the neck upon slight exertion, inability to concentrate his mind sufficiently to write his sermons and had especially bad nights after his Sunday efforts. He gave a history of two previous attacks of nervous prostration, one during his college course, which he said required him to lay off all mental work for a year or more and in which he was told he narrowly escaped brain fever. The other attack occurred three years before he consulted me and he lay by nearly a year at that time, spending his time on a farm with friends, where he enjoyed company, but had no responsibility. His present trouble began some months previous to the time I saw him and he had hoped to avert a repetition of his former experiences by taking a smaller church and using old material for his sermons for a time. But he had found the work too much for him and after taking a month's rest and trying again with no better result, he came to me for advice about giving up his church for six months or indefinitely and taking a long trip or going south for some months. His physical condition was carefully investigated, including the examination of his blood and urine and the opinion reached that his feeling of exhaustion and also his insomnia, pressure in the head and other troublesome symptoms, were due in part to a deficient elimination and in part to the memory of what he had suffered during his previous illnesses. After great care in the way of explanation and not without argument, which nearly ended in change of physicians, for his mind was firmly set in the belief that his former experiences were to be repeated, he came to see his case as I did and accepted the hygienic and dietary regulations directed. These included the free use of water, moderate use of meat, abstinence from tea, coffee and sweets and systematic out-door exercise. After about four weeks of this régime he came around one day and thanked me for my persistence in urging him the way I did against his own judgment and to say that he was convinced, in spite of the humiliation, that he had been suffering with hysteria instead of neurasthenia as he had supposed. He resumed his work and is still keeping well. No medicine was given, as he needed none, although he had been taking medicine of various sorts before consulting me.

Here it is perhaps well to note the uselessness of trying to keep such a patient going on tonics or stimulants; and also to state that as the condition we call fatigue is due to a toxic condition of the blood, brought about by the cell destruction, or tissue change, accomplished during exercise, either physical or mental, and we become rested when we have had time to eliminate the effete material, so the condition of habitual fatigue—that tired feeling—or that condition in which a person becomes fatigued upon the slightest exertion, is frequently not from the loss of strength or power, but from such insufficiency in the elimination that it only requires an exceedingly small amount of broken-down tissue products to be added to induce such a condition of saturation of the system as to produce the feeling of fatigue, while at the same time the patient be really strong. Such was the state of affairs with the last case described and it is a condition often overlooked. When the average specific gravity of the urine is over 1.024 and the blood presents 10 or 15 per cent. of concentration above the average, even if there are no signs of organic disease of kidneys or other organs, the condition deserves attention, which at that time may avert serious trouble.

In the way of a closing suggestion, it may be said that the laws of food ingestion and elimination, exercise and rest, which govern the development of a strong muscular system, are akin to, if not identical with, the laws which govern the development of a strong nervous system. And the same logic which will apply to building up a strong muscular system from an exhausted or wasted one will also apply to the restoration of a weak nervous system or a neurasthenic. We would not think of more than a few days of complete muscular rest after muscular exhaustion, for instance, and then would advise carefully graduated exercise. The same kind of management may be applied to the person whose nervous system has become exhausted from an over-strain and it will not open the way for the development of hysteria and hypochondriasis. The patient will not empty his mind of every thought that touches on his business or profession and let it remain a vacuum, but will occupy it with something, and our great problem is to furnish such a patient some occupation which will do him good and not harm.

### DISCUSSION.

Dr. Mann, of Buffalo: I do not think Dr. Loveland's paper ought to pass without a word, because it seems to me to be exceedingly important. I believe there are a great many of these cases, such as the doctor describes, that are treated entirely wrong. A great many cases come to me for operation, even for serious operations, where there is no necessity of any operation at all, where the perverted nerve functions are due to something entirely different from a local, say, uterine or pelvic condition, but where they are due more to the condition that the doctor describes than to anything else. I believe that this auto-intoxication—the doctor did not use the term, but that is the condition which he describes—that this auto-intoxication which the doctor mentions is one of the most common causes of this state which we find so frequently, which we find more commonly among women than we do among men. We can find out very quickly whether this is the case or not. The test of the urine which the doctor gave will tell the story very quickly. In my private hospital a while ago I took twenty cases, as they came into the hospital, of women suffering from various pelvic disorders and put their urine to careful examination, a twenty-four hours' sample, and tested it. I found that of twenty women the average amount of urine passed in twenty-four hours was fifteen ounces; in some of them it was down as low as four ounces. They didn't have Bright's disease; they didn't have suppression of the urine exactly; but they had what we might call a constipation of the kidneys, a condition which is just about the same as that which we find in the bowels. Their bowels were constipated, too. I found it was utterly impossible to do anything with them. I might take their insides out, sew them up and do all sorts of things with them and they would not be one bit better until I had got their eliminations into proper condition, and this consists in getting their skin to act, by hot-air baths and so on, and getting the kidneys to act, because the kidneys will act after the skin has relieved them somewhat of the overburden of poison which is put upon them. They are tired out by the amount that is brought to them. They cannot act. If you get rid of a certain amount of it by the skin they will act again; and by making them drink large quantities of water. Some of them wouldn't drink water at all; had to make them take a teaspoonful at a time. One woman said water made her sick and she couldn't take it. I told her she could go home until she could take water. One woman laid and cried three days because I told her she had to take water, teaspoonful at a time. She ended by getting well, drinking about two quarts a day. Elimination will not go on except it has something to eliminate it with and water washes out the system, helps the kidneys, helps the bowels to do their work properly. Of course, we must look into the other points to see that there is no local irritation, which is the starting point. There may be some eye trouble, displaced kidney, misplaced uterus; there may be one of a dozen different things which is the starting point of the trouble and which should be corrected, but you will do nothing towards the relief of the patient until you get their eliminative organs in proper working order.

The President, Dr. ANGELL, of Rochester: Any further discussion on this subject? I would like to emphasize, if I may, the remarks made by Dr. Mann in addition to the paper by Dr. Loveland, and I would like to say that I believe so largely in the power of thorough elimination in relieving many of these nervous symptoms that in perhaps 50 per cent., perhaps 75 per cent., of the cases of functional nervous disorder that come under my observation, I am constantly using water in large measures and also resorting to the use of salicylate of sodium or salicylates in some form, which is one of the best means we have of increasing elimination of the urates or uric acid, and I have obtained even better results by this method of treatment than by treating them symptomatologically and putting them to bed and giving them rest. Of course, in some cases we have to do that. I simply call attention to the bad influence of subjective attention. I mentioned that this morning in my paper, but I cannot emphasize it too much. If you can win people from thinking about themselves and their physical processes, you have not only done that patient good for the time being, but you have given him a liberal education.

Dr. WEIGEL, of Rochester: In line with this same subject, Dr. Loveland's paper, I want to call attention to certain classes of cases which are exceedingly common, and that is nervous breakdown in school children, a class of cases which is increasing because our school-work makes such demands, particularly upon the girls. We hardly stop to realise that when a girl reaches the age of twelve or fifteen years that the whole constitution is undergoing a physical change, which is in itself a great task upon the nervous system. If, then, you add to that the natural strain, the strain of the study, the mental application, it is not to be wondered at that sooner or later many of those children, who are very attentive to their books, will break down. In those cases I find that it is not medicine that they require, but simply to direct their energies into the proper channel or to give them an entire change of occupation. As a rule we find that those patients do not take any exercise whatever. They go to school from nine to twelve and from one to four o'clock; they take piano lessons after school and when they get through with that they have some more studies; they don't get outdoors at all. In some extreme cases of nervous breakdown that I have seen of that kind I have either stopped the attendance entirely, or very materially lessened the mental application and then put them through a course of systematic muscular exercise treatment, with recovery usually in a short time and without any drugs whatever. Mothers will bring their daughters to me and say, "this child is weak and tired out and needs a tonic." If you will simply throw your physic to the dogs, give those children physical exercise and particularly under your own personal direction, I am sure that you will get results that you cannot obtain in any other way.

